# TREM2 has a significant, gender-specific, effect on human obesity

**DOI:** 10.1038/s41598-022-27272-x

**Published:** 2023-01-10

**Authors:** Tzila Reich, Orit Adato, Naomi Schneid Kofman, Ariel Feiglin, Ron Unger

**Affiliations:** Faculty of Life Sciences, The Mina and Everard Goodman, Bar-Ilan University, 52900 Ramat Gan, Israel

**Keywords:** Computational biology and bioinformatics, Genetics

## Abstract

Triggering Receptor Expressed On Myeloid Cells 2 (TREM2) is a membrane protein expressed on immune cells, involved in neurodegenerative diseases and cancer. Recently, it was shown that TREM2 is expressed on lipid associated macrophages in adipose tissue, and that TREM2 knockout mice suffer from metabolic symptoms. Here, a computational study using public databases, brings direct evidence for the involvement of TREM2 in human obesity. First, we show a significant correlation between TREM2 expression levels and BMI in adipose tissues in samples from the GTEx database. This association was evident for males but not for females. Second, we identified in the UK Biobank cohort a coding SNP in TREM2 with a significant effect on BMI. Compared to previously identified SNPs associated with BMI, this SNP (rs2234256 SNP, L211P) has the strongest association, reflected in significantly higher BMI values of people carrying the SNP as heterozygous and even more for homozygous. Strikingly, this association was evident only for females. These observations suggest a novel gender-specific role of TREM2 in human obesity, and call for further studies to elucidate the mechanism by which this gene correlates with an obese phenotype.

## Introduction

TREM2 (Triggering Receptor Expressed On Myeloid Cells 2) is a membrane protein expressed on immune cells. The protein has been implicated in neurodegeneration, particularly in Alzheimer’s disease^1,2^.

A recent study^3^ used transcriptional single-cell sorting to show that TREM2 is also expressed on lipid-associated macrophages, found in adipose tissue. It was shown that TREM2 knockout mice suffer from metabolic symptoms including adipocyte hypertrophy, hyper-cholesterolemia, body fat accumulation, and glucose intolerance. A very recent study described in a preprint^4^ identified a novel subpopulation of macrophages expressing high level of TREM2 in the kidney of obese mice that could be matched to population of macrophages expressing high level of TREM2 in obese humans.

In recent papers^5–7^ the role of TREM2 in cancer was elucidated showing that TREM2 is a marker of tumor-associated macrophages in cancers, that high expression of macrophage TREM2 correlates with poor survival, and that blocking TREM2 enhances anti-tumor response in mice. Thus, it is becoming clear that TREM2 is a major player at the intersection of cancer, metabolic, and neurodegenerative diseases.

In spite of the recent interest in TREM2, there is little epidemiological evidence for involvement of TREM2 in human obesity. One small study based on 15 subjects with obesity and diabetes found increased expression of Trem1 and reduced Trem2 expression in several tissues and in the blood^8^. Here, we examined the association between TREM2 expression and human obesity, using two large public datasets: The Genotype-Tissue Expression (GTEx) project^9^ and the UK Biobank^10^.

## Methods

### Database downloads

Data from GTEx version 7 database were downloaded from the GTEx portal along with sample phenotype data from dbGAP, including BMI values. Spearman correlations were calculated with the R cor.test function separately for samples from each tissue.

SNP data were downloaded from UK Biobank in June 2021, while the BMI data were downloaded from UK Biobank in August 2020; there were 481,271 participants for whom both SNP and BMI data were available.

Coding SNPs in TREM2 were identified from gnomAD v3.1.2^11^. Only SNPs with allele frequency > 0.001 were considered.

The list of SNPs that are known to correlate with BMI in the European population was compiled from Table 1 and Extended Data Table 2 of Locke et al.^12^.

### Retrieval of SNPs with strong linkage disequilibrium

UK Biobank SNPs that are in strong Linkage Disequilibrium (LD) with SNPs that are known to have strong association with BMI in European population but do not appear in the UK Biobank chip, were identified using Ensembl REST APIs^13^ and GWAS Catalog REST APIs^14^. For every BMI-associated SNP, we selected all the SNPs that are in LD in the ‘British in England and Scotland (GBR)’ population, using a genomic window of ± 25 K bp. Of the resulting LD SNP list, we chose the SNP that appeared in the data downloaded from GWAS Catalog with the highest r^2^ value. Entrez Programming Utilities (eFetch and eSummary)^15^ were used to retrieve detailed SNP information (e.g. SNP position and merged SNPs) from the NCBI SNPs database.

### Comparing average BMI of different cohorts

For every SNP, the average BMI was calculated separately for male and female cohorts, and in every cohort, for wild-type individuals, for homozygous and for heterozygous for the SNP. Using two tail Student’s t-test, the average BMI of the cohort that does not carry the SNP was compared to the average BMI of cohort participants with the SNP, either homozygous or heterozygous. (*P*-value was Bonferroni adjusted to number of SNPs included in the analysis).

## Results

The GTEx project is a public resource that is used to study tissue-specific gene expression, Samples were collected from 54 non-diseased tissue sites across nearly 1000 individuals (although not all tissues have been analyzed for all individuals). The GTEx bulk tissue gene expression profile for TREM2 (https://gtexportal.org/home/gene/TREM2) shows that TREM2 is mostly expressed in the brain, lung and nerve tissues. Adipose tissues (subcutaneous and visceral) are ranked 12th and 20th respectively in terms of normalized expression level among the 54 tissue types. We calculated the Spearman correlation coefficients between TREM2 expression levels and BMI value for every tissue type. Six tissue types (out of 54) demonstrated significant (*P*-value < 0.05) correlation between TREM2 expression level and the BMI of the individual doners (Table 1). Adipose tissues (Subcutaneous and Visceral) showed the highest and most significant correlation. Performing the analysis by gender, revealed that these correlations were more significant for males than for females. Supplementary Table 1 lists the correlation and sample size for all tissues. In order to make sure that the stronger effect for man is not a result of the fact that there are more males (285) then females (157) in our sample, we performed the following experiment: We randomly chose 1000 times a subset of 100 males participants and 100 females participants and calculated the correlation between the TREM2 expression level and the BMI of the individuals. For the adipose subcutaneous tissue, the average *P*-Value for males (computed by a geometrical mean of the 1000 runs) was 0.000676 while the corresponding *P*-Value for females was 0.107, three orders of magnitude difference. For the adipose Visceral tissue, the *P*-values were 0.0031 for males and 0.035 for females. These results support our claim that the effect is significantly more evident for male than for female.

Another way to explore the connection between TREM2 and obesity, is to analyze the BMI of people with SNPs in the TREM2 gene. There are 8 coding SNPs with frequency > 0.001 in TREM2 in the gnomAD^11^ database. Out of these eight SNPs, three SNPs are included in UK Biobank (namely rs2234256, rs142232675, rs143332484). SNP rs2234256 is a missense coding SNP in TREM2, leading to a change from Leucine to Proline at amino acid 211. This SNP is not reported in Clinvar as known to have a clinical significance. We used the UK Biobank database to check the BMI of individuals with this mutation. Out of 476,884 participants, we identified 4148 heterozygotes for the mutation (0.87%) and 203 homozygotes (0.04%).

**Table 1:** Tissues with significant correlation between TREM2 expression and BMI.

Tissue	Total			Male			Female		
	Spearman Correlation	*P*-value	N	Spearman Correlation	*P*-value	N	Spearman Correlation	*P*-value	N
Adipose—Subcutaneous	0.251	9.05E−08	442	0.348	1.59E−09	285	0.136	0.08942	157
Adipose—Visceral (Omentum)	0.255	1.08E−06	355	0.279	1.11E−05	241	0.181	0.05388	114
Colon—Transverse	− 0.207	0.00057	274	− 0.200	0.01024	164	− 0.196	0.04011	110
Lung	− 0.142	0.00330	427	− 0.220	0.00018	286	0.025	0.77287	141
Brain—Caudate (basal ganglia)	− 0.191	0.01534	160	− 0.192	0.04009	115	− 0.212	0.16270	45
Pancreas	0.151	0.01709	248	0.154	0.05973	150	0.097	0.34422	98

Table 2 shows a significant effect of this SNP on BMI, 0.92 BMI units for heterozygotes and 1.72 BMI units for homozygotes. The variation of BMI in the population is obviously large (as can be seen from the standard deviations) but the large size of the cohort leads to the high statistical significance (8.2 × 10^–40^) of the difference in BMI calculated by a two-tailed Student t-test between carriers and non-carriers. Strikingly, this SNP specifically affects females, and not males. The two additional coding SNPs in TREM2 for which we had relevant data from UK Biobank, rs142232675 and rs143332484, did not show any association with BMI.

To appreciate the magnitude of the effect of the rs2234256 SNP on BMI (i.e. the increase of 0.92 BMI units for heterozygotes and 1.72 BMI units for homozygotes), we compared it with the effect of 77 SNPs that were reported to be significantly associated with BMI in the European population^12^. Out of these 77 SNPs, we were able to locate 19 SNPs directly in the UK Biobank SNPs registry. For 55 SNPs (out of the additional 58 SNPs) we identified the SNPs that are in strong LD with the SNPs of interest and appear in the UK Biobank (see Methods). There were 3 (out of 77) SNPs that were not included in the analysis due to lack of information: rs2033529, rs7141420 and rs13078960.

Supplementary Tables 2 and 3 (for the 19 and 55 SNPs respectively), show that when comparing between wild type and homozygotes, only 2 of the known SNPs (rs76828367 & rs11847697) had an effect similar to the effect of the rs2234256 SNP on BMI, and *none* had a similar effect when expressed in heterozygotes. Accordingly, considering both homozygote and heterozygote effects in the UK Biobank cohort, rs2234256 has a larger effect on BMI than any other known SNP in the European population.

**Table 2:** Average BMI of individuals with and without the rs2234256 SNP.

	Number of individuals			Average BMI			t-test		
	W.T	HZ	HM	W.T	HZ	HM	W.T. versus rs2234256 carriers	W.T. versus rs2234256 HZ	w.t. versus rs2234256 HM
Total	476,884	4184	203	27.41 ± 4.2	28.33 ± 4.3	29.13 ± 5.1	8.2 × 10^–40^	2.48 × 10^–35^	3.13 × 10^–07^
Males	218,169	1959	104	27.84 ± 5.2	27.76 ± 6.0	28.55 ± 4.6	0.45	0.708	0.084
Females	258,715	2225	99	27.06 ± 4.8	28.74 ± 5.3	29.74 ± 4.9	9.4 × 10^–58^	8.56 × 10^–53^	2.48 × 10^–07^

*W.T*. wildtype, *HZ* Heterozygous, *HM* Homozygous.

## Discussion

This study shows the involvement of TREM2 in human obesity. We describe a significant correlation between TREM2 expression level and obesity in adipose tissues in the GTEx database. This association was evident for male but not for female donors. We also identified a coding SNP in TREM2 with significant effect on BMI in the UK Biobank cohort. This SNP (rs2234256 SNP, L211P), shows the highest effect (when considering both homozygote and heterozygote effects) with increased BMI compared to 74 SNPs that were previously shown to have a significant effect on BMI. Although rs2234256 is not frequent (which is probably the reason it was not identified in previous studies) it has a non-negligible frequency in the UK biobank: 0.87% for heterozygotes 0.04% for homozygotes. Due to the size of the UK Biobank cohort, this amounts to 4148 heterozygotes and 203 homozygotes for this SNP, and thus the *P*-value of the effect was extremely significant (8.2 × 10^-40^). Strikingly, the effect on BMI was evident for females only, and was not seen in male participants.

The correlation between TREM2 expression level in adipose tissue and BMI is much stronger for males than for females, while the effect of rs2234256 SNP is evident only in females. However, the two effects reflect different phenomena, the former relating to the expression level of mostly wild type genes in adipose tissues on BMI, and the latter relating to the effect of a specific coding mutation in heterozygotic and homozygotic carriers.

This is a computational study, based on large voluntary public databases which may contain selection bias and various confounders. In addition, the correlations we have shown cannot prove causality. However, we suggest that, taken together with the experimental data in mice in^3,4^, our findings warrant additional studies investigating the role of TREM2 in human obesity, especially regarding the intriguing gender-specific effects it may exert.

Trem2 is an important gene which has been implicated to be involved in neurodegenerative diseases and cancer, understanding its role in metabolic diseases and obesity may shed light on additional biological functions of this multifaceted gene.

## Supplementary Information

Supplementary Information 1.

Supplementary Information 2.

Supplementary Information 3.

## Data Availability

The gene expression data used in this steady were downloaded from the GTEx portal and are available upon request from the corresponding author (RU). The SNP data analyzed in this study were achieved from the UK Biobank under a license to RU. These data are however available from the corresponding author (RU) upon reasonable request and with permission of the UK Biobank.
