# Author Correction: TREM2 has a significant, gender-specific, effect on human obesity

**DOI:** 10.1038/s41598-023-32425-7

**Published:** 2023-03-29

**Authors:** Tzila Reich, Orit Adato, Naomi Schneid Kofman, Ariel Feiglin, Ron Unger

**Affiliations:** Faculty of Life Sciences, The Mina and Everard Goodman, Bar-Ilan University, 52900 Ramat Gan, Israel

Correction to: *Scientific Reports* 10.1038/s41598-022-27272-x, published online 10 January 2023

The original version of this Article contained an error in the spelling of the author Ariel Feiglin which was incorrectly given as Ariel Faiglin.

The original Article has been corrected.

